# Case of Erythema Tuberculatum Et Œdematosum

**Published:** 1856-05

**Authors:** 


### RECORD OF MEDICAL SCIENCE.

### *Case of Erythema Tuberculatum et Œdematosum.*

[Read before the Boston Society for Medical Improvement, by S. Durkee, M. D.]

Patient, Mrs. Capen, of South Boston. Age, 47 years. Born of healthy parents, and always lived in Massachusetts. Was married at the age of 19, and was the mother of six children. They are all living but one, which died at the age of four months. Her mother is still living, at the age of more than 70 years. Her father died at sea of an injury.

Patient was above the medium size, stout built, dark complexion, black hair, black eyes; temperate, and otherwise of good moral character. Never had any unusual trouble connected with child-bearing, nor any catamenial derangements worth mentioning. She first consulted me about the middle of July, 1855. There was then one tubercle situated on the inside of the left leg, midway between the knee and the ankle. It was one inch in diameter at the base, and elevated one third of an inch above the surrounding skin. It was of a deep red color, was perfectly round, and slightly elastic to the touch. It was not painful. It began to show itself in the month of March previous, making about four months before I saw it. There were from eight to ten papulæ irregularly distributed in the immediate neighborhood, and varying in size from mere dots to a pin's head; and two or three small tubercles of the bigness of a split pea near the inner malleolus. The appearance of the papules and the tubercle corresponded with the description and plate which may be found under the head of Erythema papulatum et tuberculatum in the "Illustrations of Cutaneous Diseases," by Robert Willis, London, 1841.

The leg and foot were somewhat swollen and œdematous. The general health was impaired. The woman had a poor appetite; looked thin and pale; and complained of severe constipation and debility. Pulse 65 and feeble.

She stated that on the 4th of March, 1853, her right thigh was amputated in consequence of a disease on the right leg like that which occupied the left leg at the time I saw her in July. Both legs and feet had been more or less swollen, especially in warm weather, for six or seven years previously to the amputation. The tubercles on the right leg had existed eight months before the surgical operation was performed. They were fused together in one large mass, according to the patient's statement, and covered a portion of integument on the middle of the leg nearly as large as her hand. She had a favorable recovery from the operation, and for eighteen months afterwards enjoyed health. The malady never showed itself in the stump. The left leg was free from disease at the time the thigh was removed.

Such is the report furnished by the patient at my first interview with her. Circumstances beyond my control prevented me from seeing her again until

Sept. 29, 1855.—At this time I visited her with her medical attendant. Found the foot and leg more swollen and œdematous than when I saw her in July; and more than one hundred papules and tubercles were developed upon the leg. Some of them were entirely separated from others; some in close proximity at their bases, and some had coalesced into one common mass, especially in the neighborhood of the first original tubercle. This aggression of tubercles covered a surface nearly equal to the palm of my hand; and was elevated at some points one inch above the integument. Before any topical remedies had been applied, excepting the most simple, large sloughs had been cast off from this tuberculoid growth; and thus a cavity had been produced, one inch in length, one third of an inch wide, and extending beneath the base of the tubercles into the subjacent integument. There was no purulent or serous discharge from this deep opening; and the patient stated that there never had been. I learnt from the medical attendant that he had sprinkled bichloride of mercury upon portions of this large group of tubercles for the purpose of producing suppuration—an event which did not take place. He also stated that he had, with the same end in view, applied a solution of nitrate of silver, ʒiii. to ℥i., but that no suppuration had been produced. The patient stated that from the time these local means were used, the limb became more painful and the disease advanced with greater rapidity than before.

The largest isolated tubercle was imbedded in the skin just above the inner malleolus. It was more than an inch in diameter, and elevated half an inch. It had acquired this growth in about three months. The integument of the outer portion of the leg was at this date nearly free from tubercles and papulæ. The most minute specimens of the latter could be felt in the substance of the skin before they had scarcely risen above its surface. They were perfectly hard at this stage of their development, and of a bright red color. On the summit of them the intensity of the red tint was a little obscured, as if the cuticle had been thickened or partially detached from the derma. By pressing them with the point of the finger, the color disappeared, and on the removal of the pressure it was quickly restored.

The hairs had fallen out from the whole integument of the limb excepting an irregular islet or patch, about five inches in length and from two to two and a half inches in breadth, on the outer aspect of the lower part of the thigh and the upper part of the leg. Upon this district of skin most of the hairs were firmly retained in their sheaths until the patient died, although in other respects it was the seat of the same morbid changes that were displayed in its vicinity. All the follicles from which the hairs had escaped were congested just sufficiently to attract attention; and by drawing the finger over the skin many of them felt distinctly hard and solid; in others, the diseased action had been too feeble to produce such a condition, except to a very slight degree.

Generally speaking, the tubercles were from one fourth to one third of an inch in diameter when at their full development. A few specimens, however, were three fourths of an inch in diameter and half an inch above the level of the skin. They were of a dark red or purplish color, soft and elastic to the touch, and required for their maturity from eight to twelve weeks. Some of them, after they had ceased to increase in height, continued to augment by peripheral growth at the base.

One papule appeared upon the very end of the great toe. In ten days it grew as large as twice a mustard seed, after which it began to diminish, and in five or six days more was gone.*

Oct. 20, 1855.—Patient reports that there is a constant dripping of watery fluid, occasionally tinged with blood, from the deep cavity already spoken of as having been produced by the sloughing of the large tuberculoid mass; and the mass itself is flattening down and diminishing in size. No abatement of swelling or pain in the limb.

[Putrefaction, decomposition and wasting away of the tubercles, attended with a peculiar mawkish odor, slowly followed, attended with diminished exudation. The larger masses required from 8 to 10 weeks for their obliteration.]

The quantity of pus elaborated during the three months of my attendance upon the case was certainly microscopic; and ulceration, in the ordinary use of the word, did not take place. This fact, in connection with erythema tuberculatum, is mentioned by Prof. Wilson (p. 142).

Another fact to be mentioned in this connection is the condition of the epidermis. On the 10th of November the integument of the leg began to assume a dark color, as if it had been stained with a solution of nitrate of silver, although none had been used for forty days. The color continued to deepen from day to day until it became nearly black; the cuticle remained quite adherent after it had acquired its darkest hue; it could, however, be detached from the derma in small lamellæ or flakes as the parts were washed from time to time in chlorinated water. When dry, it was as thick as very stout writing-paper, and was very brittle. Under the microscope it appeared to be entirely disorganized, except where its under surface was attached to the cutis. Here a few epidermic scales were found in a normal state. On several occasions I examined the tissue now under consideration, and always found the above named-appearances. The cuticle cracked in all directions and afterwards exfoliated,—but was reproduced in a few days, and thus the leg looked as if covered with black scales.

Tubercles continued to be evolved, one after another, in pretty rapid succession, until not only one leg, but a large part of the integument of the thigh, was covered with them.

* The anatomical distribution of the vessels of the part affords an explanation of this phenomenon. A single arterial twig is divided so as to supply quite a number (twenty or thirty) of the cutaneous papillary loops with blood, which is afterwards poured into one common venous ramuscule. When these vessels, as in cases of impeded circulation, are abnormally distended with blood, the entire group of congested papillæ will present the appearance of a single minute red point; and if the disturbance of the circulation extends to the contiguous papillary groups, the red spot will be greater or less according to the number of papillæ involved in the congestion. Vide Wedl's Pathological Histology, p. 208.

Three weeks immediately preceding death, all the tubercles then existing, became very much flattened, and formed a striking contrast to the bold outlines which they presented at the time the artist was employed to take drawings of them (Oct. 11th). On and after the 22d of December, cerebral symptoms were present. Patient ceased to recognize her friends except now and then; was rather stupid, although she could be roused so as to speak a few words; no paralysis of the vocal organs; no complaint of pain or suffering; said she could see scarcely any, and it seemed to her as if it was night all the time; pupils much dilated. I frequently asked her if she knew me; she would reply in the affirmative, but almost always gave the wrong name. The first thought I had that any cerebral affection had set in, was suggested by a singularly vacant stare which she exhibited—as if her vision were imperfect.

At the *post-mortem* examination, five or six of the tubercles were about one fourth of an inch above the skin, while nearly all the rest had so far disappeared as to be scarcely perceptible above the surface, and gave to it a mere knobby or rough aspect.

In regard to treatment, I can only say it was eminently simple. From the beginning it was but too evident that nothing could be done to stay the progress of the malady; and the only course we could pursue was to study the comfort of the patient as far as possible, and to see that no injury was done by the interference of quacks and other officious persons.

The diseased limb was the only part we were allowed to examine after the death of the patient. Some of the *post-mortem* appearances have already been spoken of. *Above the knee* the skin was of a dirty livid color. Scarcely a trace of tubercles was to be seen. The only mark which indicated the spots where they had existed, consisted in the peculiar shrivelled or collapsed condition of the cuticle, from beneath which the tubercles had disappeared a few days before death. The integument of this portion of the limb was thickened to a moderate degree—say from a line to a line and a half.

*Below the knee,* the skin had a very dark reddish brown color; or a deep brown with a purplish tint. So far as relates to the mere color of this portion of the limb, a very tolerable representation of it may be seen in the London edition of Bateman's Delineations of Cutaneous Diseases, Plate xxxi.—*Boston Med. and Surg. Journ.*

